# Understanding Barriers and Facilitators to Disability and Rehabilitation Policy Implementation in Gauteng, South Africa: A Qualitative CFIR‐Informed Study

**DOI:** 10.1002/hsr2.70515

**Published:** 2025-03-04

**Authors:** Naeema Ahmad Ramadan Hussein El Kout, Sonti Pilusa, Natalie Benjamin‐Damons, Juliana Kagura

**Affiliations:** ^1^ Department of Physiotherapy School of Therapeutic Sciences, Faculty of Health Sciences The University of the Witwatersrand Johannesburg South Africa; ^2^ Division of Epidemiology and Biostatistics School of Public Health, Faculty of Health Sciences The University of the Witwatersrand Johannesburg South Africa

## Abstract

**Background:**

The effective implementation of disability and rehabilitation frameworks is essential for the full participation and social integration of individuals with disabilities. In Gauteng, South Africa, significant challenges persist in the execution of the Framework and Strategy for Disability and Rehabilitation (FSDR). This study aimed to explore the barriers and facilitators to the implementation of the FSDR in Gauteng.

**Methods:**

We employed qualitative research methods, including semi‐structured interviews and focus group discussions with stakeholders involved in the implementation process, from management to end users. Data were triangulated with a review of paper‐based implementation reports from rehabilitation managers. The interview, focus group, and document review data were analyzed using inductive thematic analysis with MAXQDA qualitative data analysis software.

**Results:**

Barriers to implementation included resource constraints, organizational inefficiencies, and socio‐cultural attitudes toward disability. Facilitators identified included inter‐sectoral collaboration, community engagement, and alignment of policies with international guidelines. Using the Consolidated Framework for Implementation Research (CFIR), barriers and facilitators were mapped to domains such as intervention characteristics, outer and inner setting factors, characteristics of individuals, and the implementation process. Key challenges included insufficient awareness and training among healthcare professionals, limited resources, safety concerns, and inconsistent policy implementation. Facilitators such as inter‐sectoral collaboration, policy development, provincial training, and advocacy by persons with disabilities were crucial for overcoming these challenges.

**Conclusion:**

This study provides valuable insights into the contextual factors influencing disability and rehabilitation policy implementation in Gauteng. By addressing identified barriers and leveraging facilitators, evidence‐based strategies can strengthen rehabilitation services, promote social inclusion, and enhance the rights of individuals with disabilities in South Africa and globally.

## 1 Introduction

Global discussions on disability and rehabilitation increasingly emphasize a rights‐based approach, prioritizing inclusive policies to promote the full participation and integration of individuals with disabilities [1]. However, translating these frameworks into practice remains challenging, particularly in middle‐income countries like South Africa, where systemic and resource constraints persist [2].

South Africa's health system reflects stark inequalities, divided into under‐resourced public and well‐funded private sectors. The public sector serves the majority of the population, exacerbating disparities in access to care [3]. To address these inequities, the government is implementing the National Health Insurance (NHI) scheme, which aims to achieve universal health coverage (UHC) by pooling resources to deliver quality healthcare based on need, irrespective of socioeconomic status [4].

Disability is a significant public health issue in South Africa due to high prevalence rates linked to injuries, chronic diseases, and congenital conditions. The [5] estimates that about 15% of the global population lives with some form of disability, a statistic mirrored in South Africa. In response, the government has developed comprehensive policy frameworks, including the White Paper on the Rights of Persons with Disabilities (WPRPD) and the Framework and Strategy for Disability and Rehabilitation (FSDR), to support individuals with disabilities and promote their inclusion [6].

The FSDR serves as a national guideline for providing comprehensive rehabilitation services, improving access to care, and fostering social inclusion. Its objectives include integrating rehabilitation across healthcare levels, enhancing service accessibility, training healthcare providers, advancing community‐based rehabilitation (CBR), and strengthening policies to protect disability rights [7]. While progress has been made, particularly in integrating services into primary healthcare and expanding CBR programs, implementation faces ongoing challenges such as resource limitations, infrastructure gaps, and insufficient trained personnel. Efforts to address these barriers include increased funding, policy reforms, and collaboration with NGOs and international organizations [8, 9].

Gauteng province, South Africa's economic hub, exemplifies these challenges within a diverse and urbanized context. With its large population and significant disparities in healthcare access, Gauteng provides a critical setting for examining the barriers and facilitators of disability and rehabilitation policy implementation. Understanding these dynamics is essential for refining policies and improving service delivery to enhance the quality of life for individuals with disabilities.

**Figure 1:**
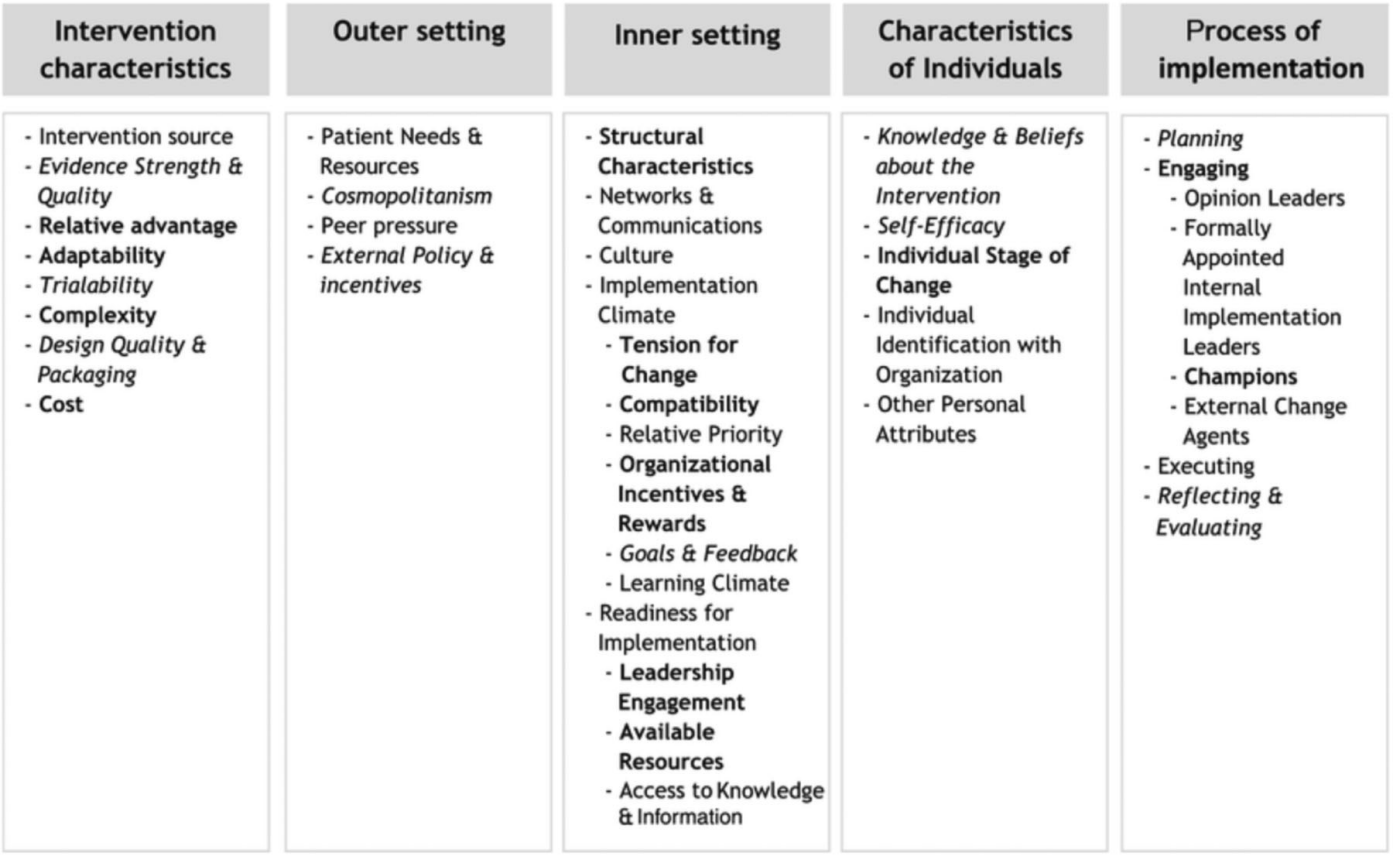
CFIR (2009) framework showing the five domains.

This study, guided by the Consolidated Framework for Implementation Research (CFIR), mapped the barriers and facilitators of FSDR implementation to domains such as intervention characteristics, contextual factors, individual roles, and the implementation process [10]. By identifying how these factors influence policy execution, the study contributes to the growing literature on disability and rehabilitation implementation science, particularly in resource‐constrained settings. The findings aim to inform evidence‐based policy recommendations and intervention strategies, strengthening disability and rehabilitation services in South Africa and beyond. The CFIR framework is shown in Figure 1 below.

## 2 Study Aim

The study aimed to explore factors influencing the implementation of the FSDR in Gauteng, South Africa, based on stakeholder experiences.

### 2.1 Methodology

This study was conducted through the South African National Department of Health and the Gauteng Provincial Department of Health, focusing on the five districts of Gauteng: City of Johannesburg, Ekurhuleni, West Rand, Sedibeng, and Tshwane. Gauteng, home to approximately 15 million people, constitutes 25% of the national population.

### 2.2 Study Design

A descriptive qualitative study design was employed, using semi‐structured interviews and focus group discussions to gather insights from key stakeholders [11]. Data collection was conducted in two phases:

- Phase 1: Reviewed the National Department of Health's (NDOH) paper‐based FSDR evaluations and analyzed provincial reports through document review.
- Phase 2: Explored stakeholder experiences via semi‐structured interviews and focus group discussions to identify factors influencing FSDR implementation. Triangulation of data sources ensured a comprehensive data set.

### 2.3 Study Participants

The study included 15 semi‐structured interviews and 6 focus group discussions. Purposive and snowball sampling were employed to ensure the inclusion of diverse perspectives, capturing a wide range of stakeholder experiences and expertise. Participants included national and provincial rehabilitation managers, disability person organizations, nongovernmental organizations, clinical rehabilitation therapists, rehabilitation professionals organizations, and academics involved in FSDR implementation. Inclusion criteria were individuals involved in the FSDR implementation at any level. Participants were excluded if they did not consent and were not involved in the FSDR implementation. Ethical clearance for the study was obtained by the University of the Witwatersrand Human Research Ethics Committee (Medical), and permission was obtained from the Gauteng Department of Health. Written informed consent was obtained for the interview and focus group discussions and a separate written consent for the audio recording.

### 2.4 Data Collection

- Phase 1: Reviewed NDOH's evaluation reports of FSDR implementation using a thematic analysis approach.
- Phase 2: Developed an interview guide with open‐ended questions, conducted interviews and focus group discussions with stakeholders, audio recorded sessions, and transcribed them verbatim. Reflexivity journals were maintained to enhance transparency and self‐awareness.

### 2.5 Data Analysis

Inductive thematic analysis, as defined by Braun and Clarke [12], was employed to identify themes in the data. MAXQDA, a qualitative data analysis software, was used to streamline coding, theme generation, and data organization, enhancing analytic rigor. An independent researcher co‐coded the reports and transcripts, with themes finalized through consensus and reviewed by two co‐investigators.

## 3 Study Findings

The study identified key barriers to FSDR implementation, including inadequate training and awareness among healthcare professionals, resource constraints, safety concerns, and disjointed policy execution. Conversely, facilitators such as inter‐sectoral collaboration, targeted policy development, provincial training, adaptive implementation strategies, and advocacy by persons with disabilities supported effective policy adoption and improved disability rehabilitation outcomes. An overview of the findings are seen in Table 1 below.

**Table 1:** Barriers and facilitators to the implementation of the FSDR in the Gauteng province.

Category	Theme	Key points
Barriers	Lack of awareness & training	Limited awareness of disability policies and inadequate training hinder effective implementation.
	Limited resources	Staffing shortages, service delays, poor infrastructure, and lack of equipment maintenance impact care quality.
	Safety concerns	High‐crime areas pose risks, disrupting service delivery and deterring healthcare providers.
	Capacity & catchment issues	Facility capacity limits, long referral times, and catchment restrictions delay access to services.
	Policy implementation challenges	Resource constraints, inconsistent implementation, and misalignment with practical needs reduce access to care.
	Departmental disconnection	Poor integration among departments creates inefficiencies and fragmented services.
	Absence of bottom‐up approach	Limited involvement of frontline stakeholders leads to impractical and less effective policies.
Facilitators	Inter‐sectoral collaboration	Partnerships across sectors and community advocacy enhance alignment and implementation.
	Policy development & engagement	Complementary policies, subnational development, and workshops ensure alignment with local needs.
	Provincial orientation & training	Orientation and training initiatives support successful policy adoption and capacity‐building.
	Flexibility in implementation	Adaptive responses, such as during COVID‐19, maintained policy goals while addressing challenges.
	Advocacy by persons with disabilities	Advocacy raises awareness and fosters equitable access to rehabilitation services.

### 3.1 Barriers to Implementation

#### 3.1.1 Training and Awareness

- Insufficient awareness and training: Healthcare professionals lack awareness and adequate training on disability policies, leaving them unprepared to align practices with policy objectives.

#### 3.1.2 Resource Constraints

- Staffing and infrastructure limitations: Insufficient staff, poor infrastructure, and lack of equipment maintenance hinder timely and quality service delivery.
- Service delays: Long waiting times and referral delays compromise patient care and satisfaction.

#### 3.1.3 Safety and Accessibility

- Safety concerns: High‐crime areas deter healthcare providers, disrupting service provision.
- Catchment area restrictions: Limited capacity and restrictive catchment areas delay access to rehabilitation services.

#### 3.1.4 Policy and Implementation Gaps

- Inconsistent and fragmented implementation: Policies suffer from resource constraints, limited integration across departments, and disjointed services, reducing their effectiveness.
- Top‐down approach: Lack of frontline stakeholder involvement creates a disconnect between policy design and practical needs.

### 3.2 Facilitators to Implementation

#### 3.2.1 Collaborative Efforts

- Cross‐sector and community collaboration: Partnerships across sectors, care levels, and community members ensure alignment with provincial needs and foster advocacy.

#### 3.2.2 Policy Development and Engagement

- Localized policy development: Subnational and complementary policies enhance relevance and implementation.
- Stakeholder engagement: Sensitization workshops and inclusive engagement strategies support effective policy adoption.

#### 3.2.3 Training and Capacity Building

- Orientation and professional training: Targeted orientation and training programs enable healthcare professionals to meet policy objectives effectively.

#### 3.2.4 Flexibility in Implementation

- Adaptation to challenges: Flexibility in responding to external factors (e.g., COVID‐19) while maintaining service quality is essential for successful implementation.

#### 3.2.5 Advocacy for Disability Rights

- Awareness and access: Advocacy by persons with disabilities raises awareness and ensures equitable access to essential services.

### 3.3 Mapping Findings to CFIR Domains

#### 3.3.1 Intervention Characteristics

- Lack of awareness and training: Insufficient awareness among healthcare professionals and inadequate training at the university level hinder effective policy implementation.
- Challenges in aligning practices with policy objectives: Misalignment due to insufficient understanding and training leads to resistance and inconsistency in implementation efforts.

#### 3.3.2 Outer Setting

- Limited resources: Staffing shortages, long waiting times, lack of equipment maintenance, and inadequate infrastructure impede effective implementation and compromise patient outcomes.

#### 3.3.3 Inner Setting

- Safety concerns: High‐crime areas pose risks, affecting staff willingness to provide services and disrupting service delivery.

#### 3.3.4 Characteristics of Individuals

- Capacity and catchment area issues: Limited capacity at district levels and long referral times hinder access to rehabilitation services.

#### 3.3.5 Process

- Policy implementation challenges: Inconsistent implementation and resource constraints undermine policy effectiveness.
- Disconnection between departments: Lack of integration results in inefficiencies and fragmented services.
- Absence of bottom‐up approach: Insufficient involvement of frontline stakeholders leads to disconnects between policy objectives and practical implementation.

Barriers and facilitators to the implementation of FSDR in Gauteng, South Africa, demonstrate the importance of addressing resource limitations, enhancing training, fostering collaboration, and ensuring policies are tailored to local needs. Advocacy, flexibility, and inter‐sectoral collaboration emerged as key facilitators. These findings provide critical insights into strengthening disability care policy implementation.

## 4 Discussion

This study examined the barriers and facilitators to implementing disability and rehabilitation frameworks in Gauteng, South Africa, using qualitative methods, including interviews, focus groups, and document analysis. It highlighted significant challenges such as resource constraints, organizational inefficiencies, and limited awareness among healthcare professionals. These barriers impede the effective delivery of rehabilitation services and exacerbate existing gaps in healthcare access for individuals with disabilities. At the same time, the study identified several facilitators, including inter‐sectoral collaboration, community engagement, and complementary policies, which could improve the implementation and sustainability of rehabilitation frameworks. These facilitators offer valuable insights into how services can be enhanced and adapted to meet the needs of individuals with disabilities.

However, the generalizability of these findings is limited, as the study focuses solely on Gauteng, which may not reflect the experiences of other regions in South Africa or beyond. Future research should explore similar barriers and facilitators in diverse geographic and sociocultural contexts to better understand the broader applicability of these findings. Additionally, the study's reliance on a “snapshot” of data restricts the ability to assess how these factors evolve over time. Longitudinal research, which examines the long‐term impacts of capacity‐building efforts and policy adaptations, would provide a more comprehensive understanding of the dynamics at play and contribute to refining future policy frameworks.

Several specific barriers to implementation were identified. The lack of awareness and training among healthcare professionals emerged as a critical issue. Smith et al. [13] found that healthcare professionals reported insufficient training in disability‐related issues during their education, leaving them ill‐prepared to address the needs of individuals with disabilities effectively. Limited awareness among healthcare workers about the FSDR content and disability‐related matters impedes policy implementation [14]. Castillo et al. [15] stress the importance of awareness in policy implementation success. Enhancing awareness through targeted training and education programs can mitigate this challenge. Research by Jones et al. [16] identified resource constraints as a significant barrier to policy implementation, resulting in disparities in access to care for individuals with disabilities. The absence of tools for monitoring and evaluating disability and rehabilitation‐related activities hamper the implementation of the FSDR [17]. Lindquist [18] emphasizes the importance of policy implementation tools, while Gómez et al. [19] highlight the challenge of developing measurable indicators for disability policies.

Tailored training programs that integrate the CFIR, particularly those addressing individual characteristics and process dynamics, would likely enhance the uptake of disability and rehabilitation policies. Another significant barrier is resource constraints, including chronic staff shortages, poor equipment maintenance, and inadequate infrastructure, which compromise the timely and effective delivery of rehabilitation services. Johnson et al. [20] highlighted staffing shortages, equipment deficiencies, and inadequate infrastructure as key barriers to delivering quality rehabilitation services. These resource constraints result in delays in treatment and compromised care quality, ultimately impeding the implementation of policies aimed at improving services for individuals with disabilities.

Clear, standardized guidelines for resource allocation and procurement, particularly for assistive devices, could help address these challenges. Effective policy implementation necessitates coordinated efforts across stakeholders. Conradie et al. [21] describe the general lack of coordinated efforts in the South African health system, reflecting challenges in FSDR implementation.

The absence of a bottom‐up approach in policy development was also noted. Engaging frontline therapists and patients in the policy development process is crucial for ensuring policies are relevant and responsive to their needs. Research by Williams et al. [22] emphasized the importance of a bottom‐up approach to policy development, highlighting the need for meaningful stakeholder engagement to inform policy decisions.

A shift toward quantifying the long‐term benefits of rehabilitation could also strengthen the case for increased investment in these services. Studies have shown that capacity constraints at the district level contribute to delays in accessing rehabilitation services, leading to patient frustration and dissatisfaction [23]. The shortage of trained rehabilitation professionals poses a significant limitation to FSDR implementation. Bettger et al. [24] note that the redirection of resources during the COVID‐19 pandemic further exacerbates manpower shortages in rehabilitation services.

Additionally, safety concerns in high‐crime areas deter healthcare providers from offering consistent care, disproportionately affecting patients with disabilities. Patel et al. [25] demonstrated that healthcare providers working in these environments face increased risks that impact their ability to deliver care safely and effectively. Safety concerns not only affect healthcare providers' willingness to work in these areas but also create additional challenges in delivering care to patients with disabilities, further impeding policy implementation efforts [26]. Finally, inconsistent policy implementation and the lack of integration between therapy departments create inefficiencies, undermining collaborative efforts [27]. Studies have shown that disjointed services and limited resource sharing between departments can create inefficiencies in service delivery and hinder collaboration among healthcare providers [28]. The use of CFIR‐based frameworks to monitor policy rollout, including practical metrics for evaluating progress, would strengthen accountability and ensure more effective implementation.

On the other hand, several facilitators of successful implementation were identified. Inter‐sectoral collaboration and community engagement were highlighted as key factors in aligning rehabilitation services with local needs. Collaboration across sectors and care levels, coupled with active community advocacy, helps ensure that rehabilitation frameworks are better tailored to the realities of individuals with disabilities [29, 30]. Inter‐sectoral collaboration is pivotal for the effective implementation of health policies. Research by Smith et al. [31] emphasizes the importance of collaboration across different sectors and systems, highlighting its role as a catalyst for policy implementation success. This collaboration extends to various levels of rehabilitation care and sectors within provinces, ensuring alignment with local capabilities and needs. Bianchi et al. [30] stress the significance of community collaboration, which fosters buy‐in from end‐users and strengthens policy implementation processes by involving community members in governance.

Longitudinal research should further explore how these collaborative approaches contribute to the sustainability of rehabilitation frameworks over time. The study also found that flexibility in policy implementation, especially demonstrated during the COVID‐19 pandemic, was crucial for maintaining services also seen by Schneider et al. [32]. The COVID‐19 pandemic highlighted the importance of flexibility in policy implementation efforts. Provinces demonstrated adaptability in response to increased restrictions and healthcare system demands, as noted by Princen et al. [33]. However, it is important to balance flexibility with the need to maintain quality. Developing metrics to evaluate the impact of adaptive strategies will be vital to ensuring consistent outcomes without compromising policy integrity. Complementary policies, such as the Gauteng Disability Rights Policy, play a crucial role in supporting broader policy implementation efforts, as highlighted by Wanzenböck and Frenken [34]. They argue that developing policies at subnational levels through consultative processes ensures alignment with local needs, potentially enhancing implementation effectiveness compared to national‐level policies.

Furthermore, advocacy and awareness‐building efforts, especially those led by persons with disabilities, play a critical role in shaping inclusive policies. Active advocacy by persons with disabilities plays a vital role in raising awareness and emphasizing the importance of disability policies like the FSDR. Their advocacy efforts contribute to highlighting the needs of vulnerable populations and ensuring equal access to essential services, as supported by various advocacy organizations and studies [35].

Future research should focus on investigating how training programs, complementary policies, and advocacy initiatives influence the long‐term sustainability of disability and rehabilitation policies. Additionally, exploring frameworks for monitoring and evaluation, such as those leveraging CFIR domains, can provide actionable insights for refining these policies and improving their implementation.

## 5 Conclusion

This study has identified key barriers to the implementation of disability and rehabilitation frameworks, including inadequate training, resource shortages, safety concerns, and inconsistent policy implementation. It also highlighted facilitators, such as inter‐sectoral collaboration, complementary policies, and community advocacy, which can drive more effective and equitable rehabilitation services. These findings underscore the urgent need to address systemic gaps to improve access to rehabilitation services for individuals with disabilities.

Policy recommendations emerging from this study include advocating for national‐level initiatives that address regional disparities, implementing participatory policy development processes, and investing in comprehensive training programs for healthcare professionals. It is also critical to develop robust monitoring and evaluation frameworks that ensure policies remain adaptable while maintaining high‐quality service delivery. Such frameworks can help identify areas for improvement and guide future policy adjustments.

The broader implications of this study emphasize the critical role of disability rights advocacy in fostering healthcare equity and improving societal outcomes. Effective implementation of rehabilitation frameworks not only enhances the well‐being of individuals with disabilities but also contributes to broader social and economic development. Prioritizing equity in rehabilitation services is essential to achieving meaningful progress in disability inclusion and healthcare delivery, which will ultimately lead to a more inclusive and sustainable society.

In conclusion, policymakers, healthcare providers, and other stakeholders must take urgent action to overcome these barriers. By leveraging community engagement and fostering inter‐sectoral collaboration, it is possible to create equitable and sustainable rehabilitation services that benefit all individuals, particularly those with disabilities.

## Author Contributions

**Naeema Ahmad Ramadan Hussein El Kout:** conceptualization, investigation, funding acquisition, writing – original draft, methodology, validation, visualization, writing – review and editing, software, formal analysis, project administration, resources, data curation. **Sonti Pilusa:** supervision, data curation, methodology, conceptualization, writing – review and editing. **Natalie Benjamin‐Damons:** data curation, supervision, methodology, conceptualization, writing – review and editing. **Juliana Kagura:** conceptualization, methodology, validation, visualization, writing – review and editing. All authors have read and approved the final version of the manuscript.

## Conflicts of Interest

The authors declare no conflicts of interest.

## Transparency Statement

The lead author, Naeema Ahmad Ramadan Hussein El Kout, affirms that this manuscript is an honest, accurate, and transparent account of the study being reported, that no important aspects of the study have been omitted, and that any discrepancies from the study as planned (and, if relevant, registered) have been explained.

## Data Availability

Data is available upon request. The data cannot be shared publicly due to ethical considerations as some information in the transcripts contains potentially identifiable information. Requests for data can be sent to the granting ethical clearance committee, The Human and Research Ethics Committee (Medical) of the University of the Witwatersrand by contacting Ms Zanele Ndlovu at Zanele.ndlovu@wits.ac.za. Naeema Ahmad Ramadan Hussein El Kout had full access to all of the data in this study and takes complete responsibility for the integrity of the data and the accuracy of the data analysis.
